# Lipid metabolism fattens up hedgehog signaling

**DOI:** 10.1186/s12915-017-0442-y

**Published:** 2017-10-26

**Authors:** Robert Blassberg, John Jacob

**Affiliations:** 1 The Francis Crick Institute, 1 Midland Road, London, NW1 1AT UK; 2 Nuffield Department of Clinical Neurosciences (NDCN), Level 6, West Wing, John Radcliffe Hospital, Headington, Oxford, OX3 9DU UK; 3 Department of Neurology, West Wing, John Radcliffe Hospital, Headington, Oxford, OX3 9DU UK; 4 Milton Keynes University Hospital, Standing Way, Eaglestone, Milton Keynes, MK6 5LD UK

## Abstract

Signaling pathways direct organogenesis, often through concentration-dependent effects on cells. The hedgehog pathway enables cells to sense and respond to hedgehog ligands, of which the best studied is sonic hedgehog. Hedgehog signaling is essential for development, proliferation, and stem cell maintenance, and it is a driver of certain cancers. Lipid metabolism has a profound influence on both hedgehog signal transduction and the properties of the ligands themselves, leading to changes in the strength of hedgehog signaling and cellular functions. Here we review the evolving understanding of the relationship between lipids and hedgehog signaling.

## Functional interactions between hedgehog signaling and lipid metabolism

**Fig. 1:**
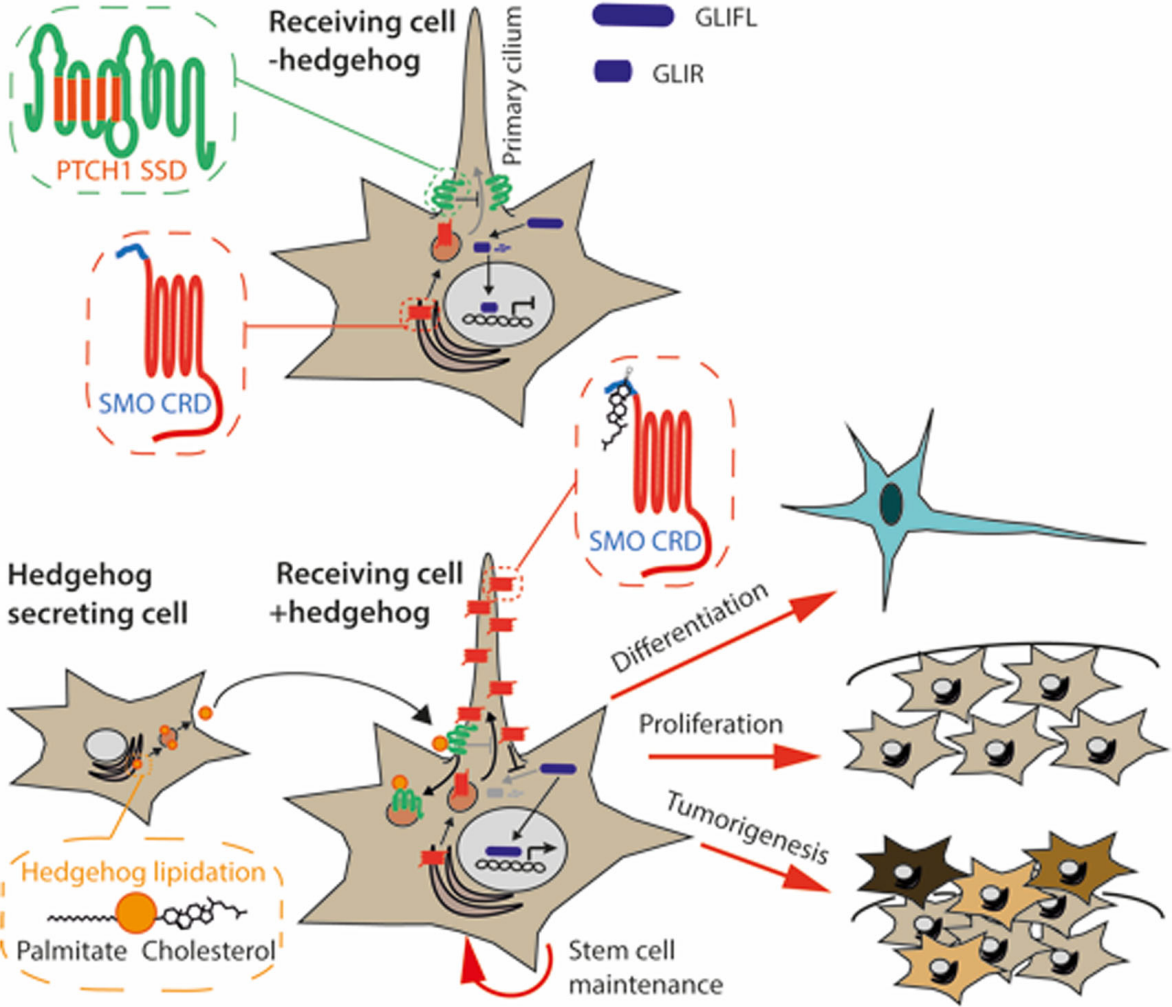
Overview of hedgehog signaling. The N- and C-termini of hedgehog proteins are covalently modified with palmitate and cholesterol, respectively. Lipid-modified hedgehog is transported by exocytic vesicles from the endoplasmic reticulum to the plasma membrane and secreted into the extracellular space. In the receiving cell, in the absence of bound hedgehog, the sterol-sensing domain (SSD)-containing receptor patched (PTCH) inhibits transport of smoothened (SMO) to the primary cilium by limiting the binding of cholesterol to the cysteine-rich domain (CRD) of SMO. In the absence of cilia-localized active SMO, GLI proteins are partially proteolyzed in the cytoplasm and translocate to the nucleus, where they act as transcriptional repressors (GLIR). Binding of hedgehog to PTCH1 leads to trafficking of PTCH1 away from the primary cilium, relieving repression of SMO ciliary accumulation and activation by cholesterol binding to the CRD. Active SMO inhibits the partial proteolysis of full-length GLI (GLIFL), which translocates to the nucleus and activates transcription. The transcriptional activity of GLI proteins drive progenitor cells along distinct differentiation trajectories. Hedgehog signaling also drives biological processes, including stem cell maintenance and progenitor proliferation. Aberrant hedgehog signaling induces aberrant proliferation and cellular differentiation associated with cancer

Hedgehog proteins are secreted ligands that enable long-range communication between cells of developing and adult tissues [1, 2]. The core molecular components of the pathway are evolutionarily conserved and were first identified in the fruit fly *Drosophila melanogaster* nearly a century ago, first through mutant analysis and later by systematic genetic screens [3, 4]. These studies elucidated the signaling mechanism by which cells sense the concentration of hedgehog in their vicinity [5], which in certain contexts can be integrated with the duration of hedgehog exposure [6]. These signal transduction events converge onto downstream gene-regulatory networks to regulate processes including cell proliferation, stem cell maintenance, survival, and fate specification [7] (Fig. 1). Many of the genes that encode hedgehog pathway components have subsequently been associated with a range of inherited human developmental disorders and other pathologies [6, 8]. The phenotype of congenital hedgehog deficiency is similar to that seen with genetic mutations causing defective cholesterol metabolism [9]. At the molecular level, three observations link hedgehog signal transduction with cholesterol biosynthesis: hedgehog ligands are covalently modified by cholesterol; the hedgehog receptor patched (PTCH) contains a sterol-sensing domain (SSD), which is found in proteins involved in cholesterol synthesis and transport; and cholesterol, its precursors, and derivatives activate or inhibit smoothened (SMO), the membrane transducer of hedgehog signaling (Fig. 1). These and other observations implicate lipids as key regulators of hedgehog signaling, which could potentially couple cellular metabolism to cell proliferation and cell fate determination. Cholesterol and phospholipids constitute the major classes of lipid and are an integral component of cellular membranes. In this review, we explore the relevance of sterols and lipids to hedgehog signaling.

## Overview of lipid synthesis

**Fig. 2:**
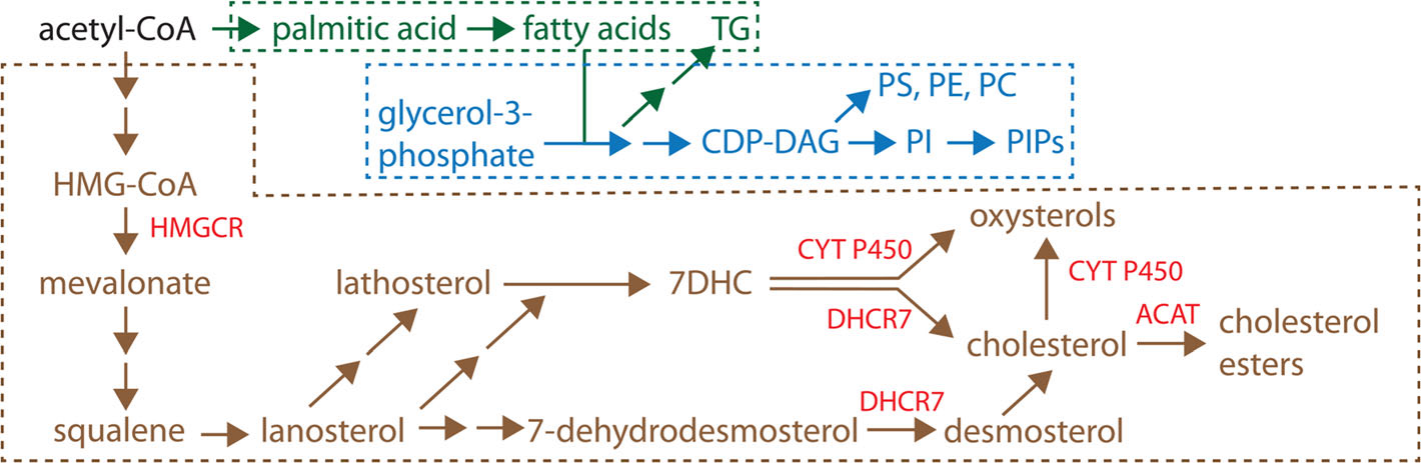
Lipid synthesis pathways. Cholesterol (*brown*), phospholipids (*blue*), and fatty acids (*green*) are synthesized from acetyl-CoA via a series of intermediate metabolites. Oxysterols are enzymatically produced from 7-dehydrocholesterol (7DHC) and cholesterol by Cytochrome P450 (CYT P450) enzyme family members, and are also generated non-enzymatically (not shown). Enzymes described in the text are shown in *red*. Both 7DHC and 7-dehydrodesmosterol are substrates for 7-dehydrocholesterol reductase (DHCR7). Fatty acids contribute to the synthesis of phospholipids from glycerol-3-phosphate. Multiple enzyme reactions not shown are represented by *double arrows. TG* triclyceride, *CDP*-*DAG* cytidine diphosphate di-acyl glycerol, *PI* phosphatidylinositol, *HMG*-*CoA* 3-hydroxy-3-methylglutaryl coenzyme A, *HMGCR* HMG-CoA reductase, *PIP* phosphatidylinositol phosphate, *PC* phosphatidyl-choline, *PE* phosphatidyl-ethanolamine, *PS* phosphatidyl-serine

Cholesterol is a tetracyclic aromatic lipid that is a major constituent of the lipid bilayers of cellular membranes. The biochemical reactions that lead to cholesterol synthesis begin with the conversion of acetyl-CoA to 3-hydroxy-3-methylglutaryl coenzyme A (HMG-CoA), which is a precursor for mevalonate (Fig. 2). Subsequently, mevalonate is metabolized to squalene via a series of isoprenoid intermediates [10–12]. Lanosterol, which is derived from squalene, is used as a substrate for the production of the cholesterol precursor 7-dehydrocholesterol (7DHC), from which cholesterol is derived by the terminal enzyme 7-dehydrocholesterol reductase (DHCR7) [13]. The other major class of lipids that constitute the membranes of the cell are phospholipids, which are generated by the enzymatic derivation of glycerol-3-phosphate with two long-chain fatty acid “tails”. Like sterols, fatty acids are derived from acetyl-CoA, which becomes elongated by recurrent esterification onto a growing aliphatic chain [14]. Fatty acid chains are subsequently modified, yielding diverse combinations of saturated and unsaturated carbon–carbon (C–C) bond arrangements [15]. The ratio of cholesterol to phospholipid, combined with the degree of saturation of phospholipid tails, determines the biophysical properties of lipid bilayers [16]. Further diversity in phospholipid classes depends on the identity of the head group linked via a phosphodiester bond to the glycerol backbone of the molecule [17]. An additional class of lipids synthesized similarly to phospholipids are the triglycerides, which are formed by the derivation of glycerol-3-phosphate with a third fatty-acid chain in place of the phosphodiester-linked head group. Unlike cholesterol and phospholipids, triglycerides are not constituents of lipid bilayer membranes and have an energy-storage function [14].

Cholesterol and its precursor 7DHC undergo sterol side chain oxidation, which generates a diverse class of bioactive sterols termed oxysterols. Depending on their specific chemical identity, these sterols regulate cellular physiology via interactions with signaling pathways, intracellular trafficking, and metabolism [18]. The signaling activities of oxysterols regulate cellular differentiation [19] and inflammation [20, 21], and they have been associated with pathologies such as atheroma [22] and macular degeneration [23]. Sterol hydroxylases, most of which are members of the cytochrome P450 family, catalyze the formation of specific oxysterol species [24]. Sterol hydroxylase-deficient mouse models have proved valuable in dissecting the specific physiological activities of several oxysterols [20, 25]. However, a major route to oxysterol formation is driven by non-enzymatic free-radical and lipid peroxide “auto-oxidation” [24, 26], confounding systematic genetic analysis of the physiological activities associated with the oxysterol metabolome. Furthermore, the low abundance of oxysterols within biological tissues compared with their precursors, coupled with the propensity for precursor auto-oxidation during sample preparation, represents a further challenge to the accurate characterization of oxysterol metabolomes [27].

## Cholesterol trafficking and homeostasis

Sterol homeostasis is maintained by feedback control at transcriptional and post-transcriptional levels across a network of diverse cellular processes. As major components of cellular membranes, sterols are transported between organelles by two analogous intracellular trafficking processes [28]. Endocytosis redistributes lipids resident within the plasma membrane (PM) via endocytic vesicles that form by PM budding and internalization. Conversely, exocytosis redistributes the lipids resident within the membranes of the endoplasmic reticulum (ER) and Golgi as constituents of exocytic vesicles that move to the periphery of the cell and fuse with the PM. Endocytic and exocytic vesicles are actively transported along microtubules by the motor proteins dynein and kinesin [29]. The direction and destination of a vesicle depends on the motor protein with which it is associated, and their selection is determined by Rab proteins localized to the endosomal membrane. Rab proteins act as molecular switches to regulate vesicular transport [29, 30]. Cholesterol modifies these interactions, influencing the flux of membranes and thus its own redistribution within the cell [31, 32]. Non-vesicular mechanisms of cholesterol transport can also shift cholesterol to various target membranes, including the PM, in a process that involves lipid-binding proteins, including caveolin 1, oxysterol binding protein-related proteins (ORPs), and proteins that contain START domains [33].

Cholesterol and its oxysterol derivatives regulate lipid and vesicular transport processes via oxysterol binding proteins (OSBPs) localized at the Golgi–ER interface [18]. OSBPs act as a tether between the Golgi and ER membranes and transfer cholesterol and phosphatidylinositol-4-phosphate (PI(4)P) between them in a process that is sensitive to the cholesterol content of these membranes [34]. Like cholesterol, PI(4)P and the related lipid PI(3)P regulate intracellular transport by mediating the interaction of vesicles with microtubule-associated motor proteins [35]. The cholesterol-dependent regulation of PI(4)P localization by OSBPs highlights an additional interaction between vesicular transport and cholesterol abundance. As this transporter activity of OSBPs is negatively regulated by oxysterols, such as 25-hydroxycholesterol (25-OHC) [34], it is evident that derivatives of cholesterol exert feedback control on cholesterol-regulated cellular processes.

Cholesterol localization is further controlled by the cholesterol transporters Niemann-Pick C1 (NPC1) and NPC2, which mobilize cholesterol from endosomal membranes [36, 37]. Mutation of the genes encoding either of the NPC proteins results in Niemann-Pick disease, which is characterized by cholesterol accumulation within the endosomal system [38]. The NPC1 cholesterol transporter belongs to a family of proteins that contain an evolutionarily conserved cholesterol-binding SSD. The SSD is a membrane-spanning motif composed of five transmembrane segments that regulates protein distribution, conformation, and activity in response to local sterol concentration [39]. Although the transmembrane segments are exposed to the lipid bilayer, permitting potential interactions with embedded sterols, physical interaction between the SSD and cholesterol has not been directly demonstrated. Rather, cholesterol and the oxysterol 25-OHC interact with an N-terminal domain common to NPC1 and NPC2 that is not membrane associated [40].

The enzymes that metabolize sterols are regulatory proteins that sense sterol levels and balance their rate of production and utilization. The activity of HMG-CoA reductase (HMGCR), which catalyzes the synthesis of mevalonate within the ER and is rate-limiting, is negatively regulated by products of the mevalonate biosynthetic pathway [41]. Rising concentrations of these metabolites promote the interaction between HMGCR and the ER membrane-associated insulin-induced gene 1 protein (INSIG1) and INSIG2 proteins via a process mediated by the SSD of HMGCR [42, 43]. This interaction increases the rate of HMGCR ubiquitination and subsequent proteolysis, thereby reducing the level of the rate-limiting enzyme in response to rising sterol levels [44, 45].

Sterols also negatively regulate the transcription of key lipid biosynthetic enzymes via the cholesterol sensor sterol regulatory element-binding protein cleavage-activating protein (SCAP). Like HMGCR, SCAP contains an SSD and is localized to the ER membrane, where it regulates the activity of the ER membrane-anchored sterol regulatory element-binding protein (SREBP) family of transcriptional regulators [46, 47]. In a similar manner to HMGCR, the SCAP–SREBP complex interaction with INSIG proteins is also stabilized by both cholesterol and its oxysterol derivatives [48]. Falling cholesterol levels lead to destabilization of the interaction between INSIG and SCAP–SREBP and to vesicular transport of the SCAP–SREBP complex to the Golgi where proteolytic cleavage of the SREBP membrane anchor occurs, releasing it to translocate to the nucleus and activate target gene expression [49]. SREBP proteins regulate the expression of enzymes participating in the biosynthesis of distinct lipid classes. Whereas SREBP1a and SREBP1c regulate enzymes involved in fatty acid and triglyceride synthesis, sterol synthesis is controlled by SREBP2 via its induction of multiple sterol enzyme-encoding genes including HMG-CoA synthase, HMGCR, squalene synthase, lanosterol synthase, and DHCR7 [50, 51]. Therefore, diverse sterol products negatively regulate the rate of biosynthesis of both steroid and non-steroid lipids via feedback inhibition as their levels rise.

**Fig. 3:**
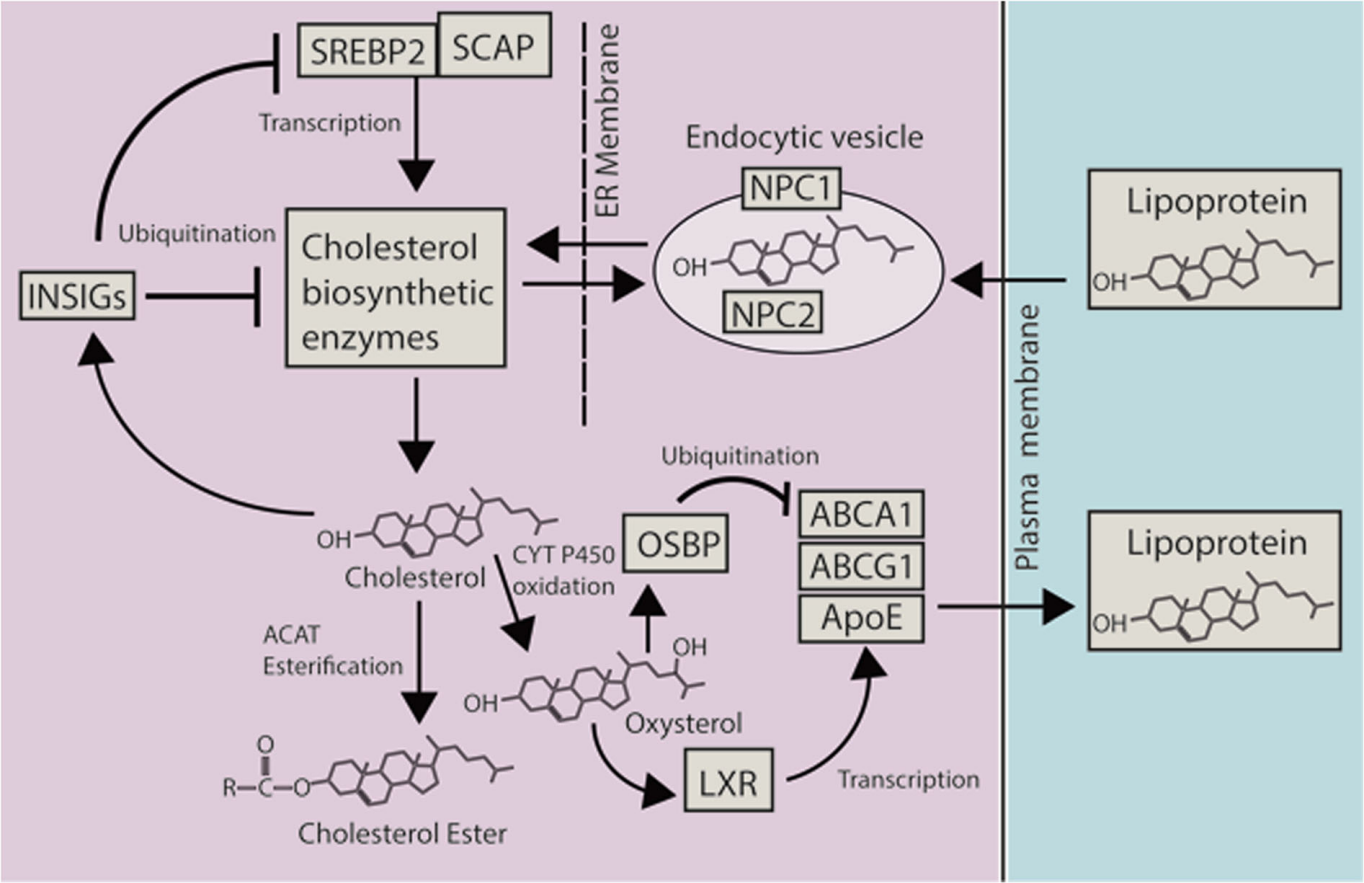
Cholesterol homeostasis is maintained by a highly interconnected network of cellular processes. The transcription factor sterol regulatory element-binding protein 2 (*SREBP2*) positively regulates the expression of cholesterol biosynthetic enzymes. Rising cholesterol levels reduce the rate of cholesterol biosynthesis by modulating the activities of insulin-induced gene (*INSIG*) proteins. When activated, INSIG both promotes the ubiquitination and consequent destabilization of key metabolic enzymes and inhibits the transcriptional activity of SREBP2 by retaining it in complex with sterol regulatory element-binding protein cleavage-activating protein (*SCAP*) in the endoplasmic reticulum (*ER*). Rising cholesterol levels also allosterically activate acyl-coenzyme A:cholesterol acyltransferase (*ACAT*), which esterifies cholesterol leading to its sequestration in cytosolic lipid droplets. Oxysterol products of cholesterol activate liver-X receptor (*LXR*) transcription factors, which positively regulate the transcription of proteins that drive cholesterol efflux from the cell (*ABCA1* and *ABCG1*), and sequester it in lipoprotein particles in the circulatory system (*ApoE*). Activation of oxysterol binding proteins (*OSBP*) by oxysterols negatively regulates cholesterol efflux by promoting ABCA1 ubiquitination and degradation. Lipoprotein-derived cholesterol is internalized in endosomes that contain Niemann-Pick C1 (*NPC1*) and NPC2, which act together to redistribute cholesterol to the ER. NPC1 and NPC2 are also critical for the egress of endogenously synthesized cholesterol from endosomes

In response to rising intracellular sterol levels, cells not only reduce cholesterol synthesis but also convert cholesterol into its storage form as cytoplasmic lipid droplets, through esterification by the enzyme acyl-coenzyme A:cholesterol acyltransferase (ACAT) within the ER [28]. ACAT undergoes positive allosteric regulation specifically by cholesterol, which it esterifies preferentially over oxysterol substrates [52–54]. In short, rising free cholesterol levels increase the rate of cholesterol sequestration by ACAT (and to a lesser extent oxysterol sequestration), whereas oxysterol levels do not affect the rate of sequestration of either sterol species. However, rising levels of certain oxysterols provide negative feedback on the levels of cellular sterols via the activation of the liver-X receptors (LXRs) [55]. LXR-α and LXR-β are orphan nuclear receptors that form heterodimers with retinoid receptors [56] and regulate the expression of the cholesterol transporters ATP binding cassette subfamily A member 1 (ABCA1) and ATP binding cassette subfamily G member 1 (ABCG1), and the cholesterol carrier apolipoprotein E (ApoE) [57], which together promote the efflux of cholesterol from the cell and its sequestration as low-density or high-density lipoproteins in the circulatory system. Oxysterol-activated OSBP promotes the ubiquitination and degradation of ABCA1 [58], reducing the rate of cholesterol efflux and providing a further example of a negative feedback process ensuring cellular sterol homeostasis. Taken together, these findings highlight that, in addition to both transcriptional and post-translation regulation of sterol biosynthetic enzymes, cholesterol and oxysterols also exert feedback regulation of enzymes and transport proteins controlling their distribution within, and redistribution from, the cell (Fig. 3).

## Inborn errors of cholesterol metabolism

Diseases of cholesterol synthesis manifest at birth or during infancy and belong to a class of genetically determined disorders termed inborn errors of metabolism. Three autosomal recessive disorders of the post-squalene pathway—Smith-Lemli-Opitz syndrome (SLOS), lathosterolosis, and desmosterolosis—are unusual in comparison to most other inborn errors of metabolism because of their association with characteristic morphological abnormalities that affect proper formation of tissues in the midline of the head [9]. The spectrum of anatomical defects observed are termed holoprosencephaly (HPE). Collectively, HPE abnormalities are the commonest abnormality of the head and face in humans, with a frequency of 1 in 10,000 births and 1 in 250 conceptions [59]. HPE does not affect closure of the neural tube, which is the commonest congenital malformation. In the most severe form of HPE (alobar HPE) the brain is small, the cerebral hemispheres are fused around a single ventricle, and the eye field fails to separate, resulting in a single, cyclopic eye. A trunk-like structure, or proboscis, is found above the single eye. In milder forms (semilobar and lobar HPE) the brain is larger and the hemispheres are partially separated. Facial abnormalities in milder cases take a variety of forms and include reduced separation of the eyes (hypotelorism), a single central maxillary incisor, and cleft lip/palate.

All three disorders of the post-squalene pathway are characterized by the accumulation of sterol intermediates, with or without a deficiency of cholesterol [9, 60, 61]. In SLOS, mutations in *DHCR7* result in a deficiency of cholesterol and accumulation of the precursor sterol, 7DHC. Lathosterolosis is caused by a mutation in the sterol C5-desaturase-like gene, *SC5DL*. Defects in 3β-hydroxysterol-24-reductase, due to mutation in the desmosterol reductase gene (*DHCR24*), result in desmosterolosis; biochemically, there is a failure to convert desmosterol to cholesterol. SLOS is by far the commonest disorder of cholesterogenesis. The rarity of lathosterolosis and desmosterolosis has meant that the phenotype of these diseases is incompletely delineated, but in broad terms all three disorders result in morphological defects on the HPE spectrum. The range and severity of phenotypic abnormalities in SLOS are highly variable. Numerous mutations have been identified in several hundred affected individuals, and although some genotype–phenotype correlations have been reported, exceptions are often identified [62].

## Hedgehog signaling

Loss-of-function mutations in the hedgehog pathway also produce HPE morphological abnormalities, suggesting a regulatory relationship between sterol metabolism and the hedgehog signaling pathway during development [63]. In familial forms of HPE, dominant loss-of-function mutations in the gene encoding the human orthologue of sonic hedgehog (*SHH*) are the most frequent genetic finding [64].

### Ligands

Higher vertebrates have three hedgehog pathway ligands—desert hedgehog (DHH), indian hedgehog (IHH), and SHH—of which SHH is the best studied [2, 65]. Hedgehog proteins are synthesized as inactive pro-peptides, which subsequently undergo cleavage leading to covalent attachment of a cholesterol molecule to the C-terminal amino acid of the active peptide [66]. Cholesterol modification reduces the solubility and diffusion of SHH, allowing incorporation into cellular membranes [67]. The N-terminal amino acid of the SHH protein is also covalently attached to the lipid palmitate by the enzyme hedgehog acyltransferase (HHAT) (Skinny Hedgehog in *Drosophila*), which is required for full activity of the ligand and, like cholesterol, alters its diffusion properties [68–71]. Lipid-modified hedgehog proteins are actively transported across the PM for release extracellularly. The translocation of SHH requires the activity of the transmembrane receptor dispatched (DISP) [72–74]. DISP contains an SSD in common with the sterol sensor SCAP and other proteins involved in cholesterol homeostasis, and it is homologous to the cholesterol transporter NPC1 [39]. Extracellular release and subsequent spreading of cholesterol-modified SHH is enhanced by its interaction with the secreted protein SCUBE2, which was first implicated in hedgehog signaling in zebrafish [75–77]. Both DISP and SCUBE2 directly interact with distinct structural aspects of the cholesterol moiety of SHH, which probably increases its solubility [78] in a manner similar to the transfer of cholesterol between NPC1 and NPC2 in endosomal membranes [79]. By comparison, in *Drosophila* the spread of hedgehog is influenced by the association of cholesterol-modified hedgehog with lipophorin particles in the hemolymph, which are analogous to the circulating lipoproteins in mammals [80].

Cholesterol-modified SHH is also shed from the surface of producing cells as a component of exovesicles or “exosomes” derived from the budding of cellular membranes [81–83]. In *Drosophila*, endocytosis and subsequent recycling of PM-associated hedgehog is required for its long-range activity and depends on the cholesterol moiety [84]. Exosomal transport of hedgehog can occur via filopodial PM protrusions termed cytonemes [82], which might also directly associate with SHH to enable long-range signaling within developing tissues [85, 86]. Finally, the formation of large multimeric complexes of SHH depends on the addition of the cholesterol moiety. As is the case for the SHH–SCUBE2 complex, formation of these multimers increases the solubility and range of spread of hedgehog within tissues, likely owing to the self-association and sequestration of the hydrophobic cholesterol moiety within the core of the complex [87–89]. Therefore, although the cholesterol adduct potentially limits the diffusibility of SHH by anchoring it to membranes [67], its critical role in mediating interactions with other molecules and multimer formation has the opposite effect, extending the range and activity of hedgehog within tissues.

### SHH signal transduction

Genetic analysis in mice and chicks demonstrated that the reception and transduction of the SHH signal in the cytoplasmic compartment of receiving cells is localized to the primary cilium [3, 90]. This organelle is an antennae-like projection of the PM surrounding a microtubule core, which is anchored at the basal body—a structure that is derived from the mother centriole. Owing to its intimate association with the centrosome, the primary cilium is dynamically assembled and disassembled over the course of the cell cycle, via processes mediated by Rab proteins [91, 92].

The mammalian SHH receptor PTCH1 is a transmembrane protein which is localized to the primary cilium in the absence of SHH [93]. Similar to SCAP, DISP, and the NPC1 cholesterol transporter, PTCH1 contains an SSD [39]. Similarities with DISP suggest that the SSD might mediate the interaction with the cholesterol moiety of SHH, but this is unclear. In vertebrates, the transmembrane proteins cell adhesion molecule-related downregulated by oncogenes (CDO), brother of CDO (BOC), and the GPI-anchored protein, growth arrest specific protein 1 (GAS1) also act as SHH receptors and form a complex with PTCH1 [94, 95]. These SHH receptors have overlapping activities that promote signaling, potentially by presenting SHH to PTCH1 [96]. Hedgehog-interacting protein (HHIP) also binds vertebrate hedgehog proteins but inhibits rather than promotes signaling, and does not physically interact with PTCH1 [97]. Homologues of CDO and BOC termed Ihog and Brother of Ihog (Boi), respectively, had earlier been identified in *Drosophila* [94]. The lipid modification of Hedgehog proteins could facilitate their simultaneous interaction with this set of cognate binding partners that modulate ligand potency [87].

In mammals, PTCH1 prevents SMO, a membrane localized GPCR-like SHH signal transducer, from entering the primary cilium. When PTCH1 binds to SHH, repression of SMO is relieved, and SMO enters the cilium where a second activating step initiates downstream signaling [93, 98, 99]. In this two-step model of mammalian SMO activation, the translocation of SMO is regarded as a prerequisite for signal transduction [100]. By contrast, *Drosophila* cells lack primary cilia, and PTCH instead regulates the accumulation of SMO at the PM, where it activates signaling [101, 102]. A non-cell autonomous model of SMO repression by PTCH has also been proposed, which could be mediated by the cholesterol precursor 7DHC [103, 104].

## Instructive and permissive effects of sterols on SHH signaling

Inhibition of SMO by PTCH1 can be overcome by cholesterol- and 7DHC-derived oxysterols [105–108]. Endogenous and synthetic oxysterols allosterically activate SMO through their binding to the extracellular cysteine-rich domain (CRD) [106, 108–110]. By contrast, the plant-derived sterol cyclopamine inhibits SMO upon binding to the transmembrane domain (TMD) at a site remote from the CRD [111]. The synthetic SMO agonist SAG competes with cyclopamine for binding at the TMD and drives SMO cilia localization and activation independently of SHH [111]. However, a B-ring oxysterol derivative of 7DHC, 3β,5α-dihydroxycholest-7-en-6-one (DHCEO), was reported to inhibit SMO by binding to a site distinct from both the CRD and the cyclopamine/SAG pocket [112]. Therefore, SMO has multiple sterol-interacting interfaces that positively or negatively affect its activity.

Cholesterol is sufficient to stimulate SHH signaling independently of oxysterols and can induce neural cell types in vitro that require moderate to high levels of SHH signaling for their differentiation [113]. Cholesterol and oxysterols compete for the same binding site in the SMO CRD [114, 115]. Furthermore, a modified version of cholesterol that cannot be metabolized to oxysterols nevertheless rescued SHH signaling in sterol-depleted cells, suggesting that cholesterol is an endogenous activator of SMO [113, 115]. As with SHH, SMO is covalently bound to cholesterol via an aspartic acid residue (Asp95) in the CRD, and its mutation results in loss of SMO cilia activation in vitro and in vivo [116].

In animal models of SLOS, the response to hedgehog signaling is reduced in receiving cells, which is consistent with a requirement for cholesterol in signal transduction [117, 118]; these models also show HPE dysmorphology characteristic of SHH deficiency. However, distinguishing between precursor accumulation versus cholesterol deficiency as the reason for attenuated hedgehog signaling has proved difficult to resolve because of the complex feedback mechanisms in the cholesterol synthesis pathway described above [119, 120]. Reduced cholesterol levels have been proposed to disrupt SMO indirectly, through an undefined mechanism involving SCAP–SREBP2-mediated transcriptional upregulation of DHCR7 [121, 122]. However, we did not find evidence to support this model in our study [118]. 7DHC and DHCEO accumulate in the brain tissue of SLOS-model animals [119, 120], but we found that 7DHC levels did not affect SHH signaling [118]. Furthermore, in SLOS-mutant fibroblasts that accumulate 7DHC and presumably DHCEO, cholesterol supplementation was sufficient to rescue SHH signaling [118]. These findings warrant further analysis of the relative contribution of 7DHC, DHCEO, and cholesterol to reduced SHH signaling in SLOS.

**Fig. 4:**
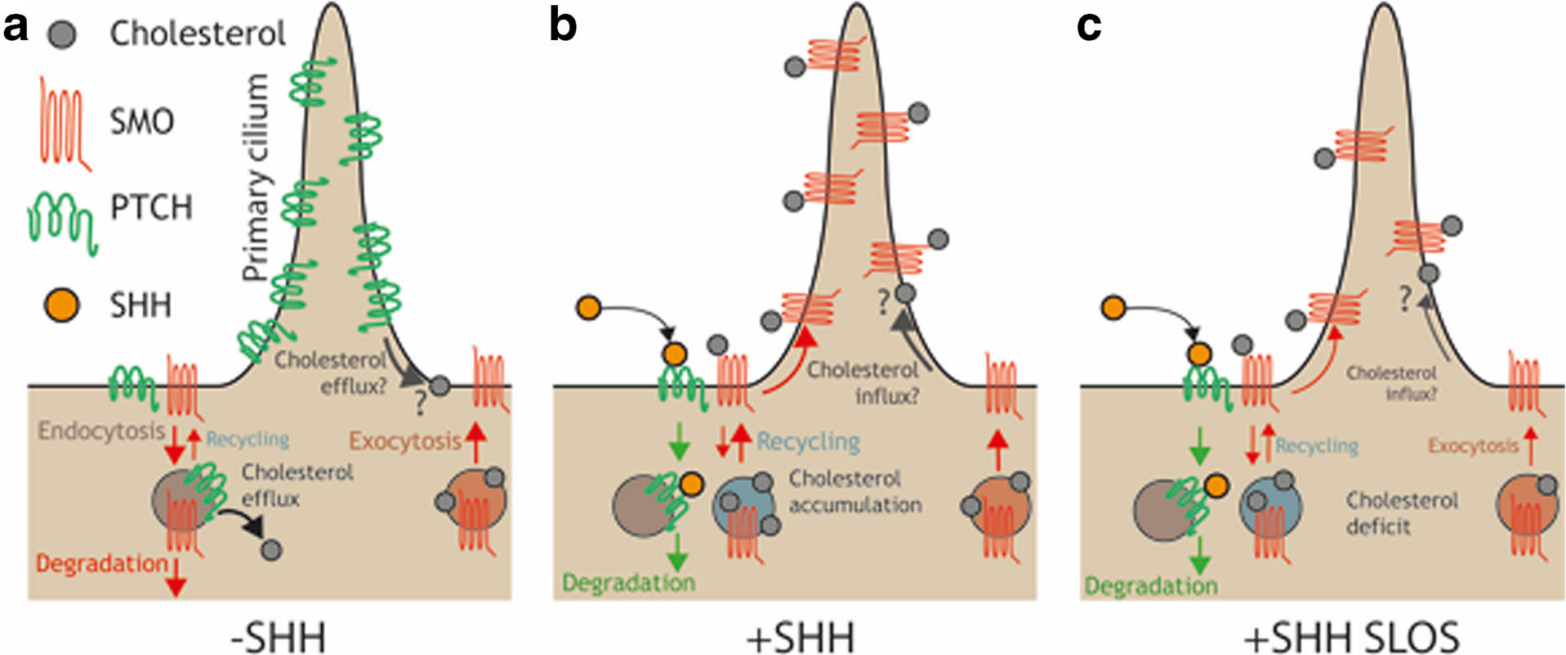
The regulation of smoothened trafficking by cholesterol. **a** The levels of transmembrane receptors such as smoothened (*SMO*) at the plasma membrane are determined by the balance between the rates of supply by exocytosis and internalization by endocytosis. Following endocytosis, receptors are either recycled back to the membrane or degraded [29]. In the absence of sonic hedgehog (*SHH*), patched1 (*PTCH1*) reduces the concentration of cholesterol in the membranes of endosomal vesicles, biasing plasma membrane SMO toward internalization and degradation [101, 139]. **b** SHH binding redistributes PTCH1 from the primary cilium and SMO-containing endosomal vesicles [93, 141]. In the absence of PTCH1, the concentration of cholesterol within endosomal membranes is elevated [139]. Elevated cholesterol levels within endosomal membranes allow SMO to accumulate in the plasma membrane by reducing its rate of internalization and degradation [101, 139]. Whether PTCH1 activity also modulates cholesterol levels in the membranes around the base or within the shaft of the primary cilium remains to be determined. **c** In Smith-Lemli-Opitz syndrome (*SLOS*), the associated reduced cellular cholesterol levels decrease the accumulation of SMO in the cilium in response to SHH [118]. This may be due to a requirement for direct molecular interaction between cholesterol and SMO for SMO cilia entry. Alternatively, reduced cholesterol levels may indirectly decrease SMO levels in the cilium by disrupting the kinetics of endocytic, recycling, or exocytic vesicle trafficking to and from the plasma membrane

The reduced SHH signaling response in SLOS might be due to an additional permissive role for cholesterol, which does not depend on the oxysterol-binding CRD or on residues in the SMO TMD that are important for the binding of cyclopamine and synthetic agonists [108, 113, 117, 118, 123–126]. Such a putative mechanism could stem from the effects of cholesterol on SMO vesicular trafficking. SMO translocation is also dependent on vesicular transport, which is influenced by cholesterol. Following its synthesis in the ER and maturation through the Golgi, SMO is trafficked to the PM. The PM pool of SMO is then internalized into the endocytic system, and SMO entry into the primary cilium has been shown to occur both by direct lateral transport from the PM and from the endocytic system [98, 99]. While SMO stimulation is generally associated with its cilia localization, these events can be dissociated by SMO inhibitors and point to distinct regulatory steps in SMO activation as described earlier [100]. Perturbations in the synthesis or subcellular localization of cholesterol alters the activities of many Rab proteins and consequently disrupts endosome motility [31, 127–129]. Altered cholesterol levels within specific cellular compartments resulting from inborn errors of sterol metabolism might therefore impair SMO cilia localization and activation owing to abnormal vesicular trafficking (Fig. 4). In support of this conjecture, Rab8 and Rab23 have been shown to modulate the rates of active SMO entry into and recycling from the cilium, respectively [130]. While the function of Rab23 with respect to intracellular trafficking is relatively uncharacterized, Rab23 is a known negative regulator of SHH signaling and is localized to both the PM and endosomal system [131, 132]. Furthermore, Rab8 is a mediator of both cilium biogenesis and cholesterol transport to the PM [133, 134]. Therefore, both Rab proteins represent plausible links between SMO trafficking to the cilium and intracellular cholesterol levels.

Compared with manipulations whereby bulk sterol is depleted, reduced SMO activation owing to DHCR7 loss of function is associated with a modest reduction in total cellular cholesterol levels [117, 118]. As DHCR7 synthesis of cholesterol is localized to the ER, cholesterol levels might be more acutely reduced in the ER and cilium-associated Golgi compartments, and thus potentially impair endosomal transport and SMO trafficking to the cilium more severely than would be predicted from bulk sterol measurements. Consistent with this hypothesis, we found that in embryonic fibroblasts from SLOS mutant mice, there was a marked reduction in SMO translocation to the cilium in response to SHH, which could be rescued by cholesterol supplementation [118]. Nevertheless, cholesterol can induce significant SMO activity that is comparable to the effect of SAG stimulation, without producing appreciable cilia localization [113]. This implies the current two-step model of SMO activation requires refinement [100].

## Lipid involvement in PTCH regulation of SMO

PTCH1 belongs to the resistance-nodulation-division (RND) family of small-molecule pumps [135]. Repression of SMO by PTCH1 occurs indirectly and acts non-stoichiometrically, implying a catalytic mechanism [136]. The homology of PTCH1 with sterol sensors involved in cholesterol homeostasis and its ability to bind and transport cholesterol have led to the proposal that PTCH1 may directly mediate the transport of cholesterol between cellular membranes [123, 136]. Whereas the residues of the SSD homologous between PTCH1 and the sterol sensor of SCAP are not essential for vertebrate PTCH1 activity, they are required for the function of *Drosophila* PTCH, limiting structure–function comparisons between the two receptors [136–138]. Furthermore, local reduction of cholesterol levels in early endosomes by PTCH has been demonstrated in *Drosophila* cells [139]. In an analogous manner, vertebrate PTCH1 might restrict access of cholesterol to SMO at the base of the cilium, which is relieved upon SHH binding to PTCH1 [113]. It is noteworthy, however, that engineered forms of SMO lacking the CRD remain partially sensitive to PTCH1 repression, suggesting an additional CRD-independent mode of SMO regulation by PTCH1 [108, 109, 136]. In addition, mutations in the SMO TMD that prevent binding of cyclopamine or its synthetic analog GDC-0449 do not affect inhibition of SMO by PTCH1 [108, 140], further indicating that PTCH1 does not repress SMO via the TMD.

Experiments in which hedgehog signaling proteins are overexpressed in mammalian cells have demonstrated the localization of both PTCH1 and SMO in endosomes, from which SMO is recycled back to the PM for entry into the cilium and activation [98, 99, 141]. In *Drosophila*, the interaction between PTCH and SMO has also been shown to occur within endosomes and depends upon a lipid molecule associated with lipoprotein particles [139]. It has been proposed that, in *Drosophila*, PTCH regulates SMO activation by controlling the distribution of the phospholipid PI(4)P and its synthesis by phosphatidylinositol 4-kinase III alpha (PI4III kinase α) [142, 143]. In this model, PI(4)P binding to the SMO intracellular domain (ICD) is critical for SMO activation [143]. Whether mammalian SMO is regulated by a similar mechanism [143] needs be tested by in vivo deletion of PI4III kinase α. Taken together, these studies raise the possibility of co-regulation of distinct SMO domains by different lipid species, cholesterol and phospholipids, and might explain why residual repression of SMO by PTCH1 occurs in the absence of the SMO CRD [108, 109, 136].

**Fig. 5:**
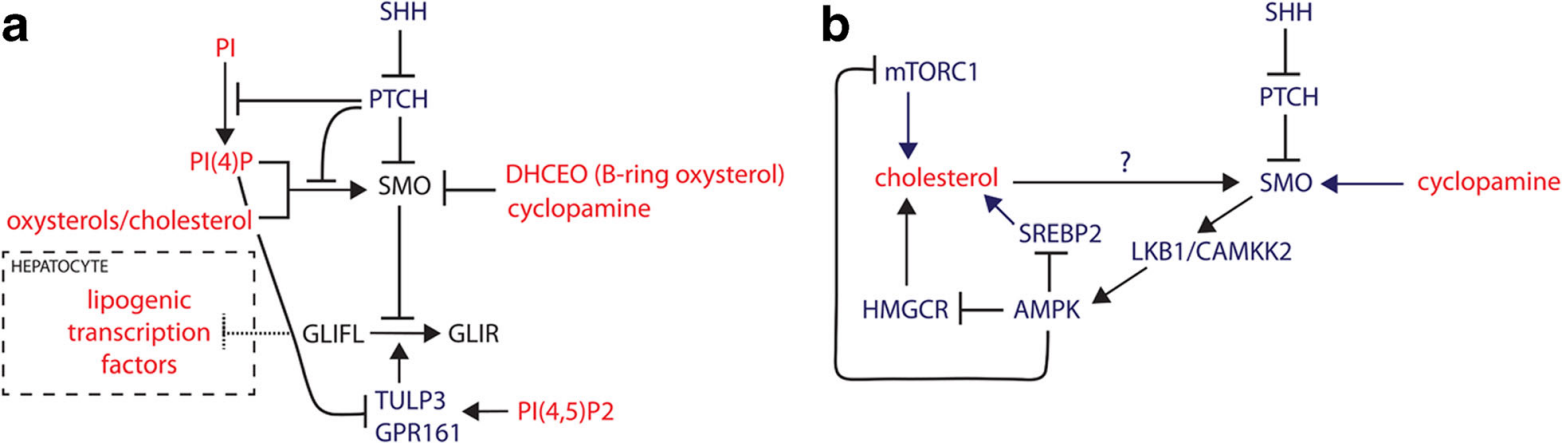
The relationship between lipids and hedgehog signaling. **a** Regulation of canonical hedgehog signaling by lipids. Smoothened (*SMO*) is the nexus of hedgehog pathway regulation by lipids. Cholesterol, oxysterols, and phosphatidylinositol-4-phosphate (*PI*(*4*)*P*) are SMO activators, whereas the plant sterol cyclopamine and a distinct class of B-ring oxysterols, DHCEO (7DHC, 3β,5α-dihydroxycholest-7-en-6-one) inhibit SMO. PTCH prevents activation of hedgehog signaling by restricting the access of cholesterol and PI(4)P to SMO while also inhibiting the synthesis of the latter lipid molecule. PI(4)P also prevents the entry of negative regulators of hedgehog signaling, TULP3 and GPR161, to the primary cilium. In hepatocytes, downstream of SMO full-length activated GLI was reported to repress the lipogenic transcriptional program (*dashed inhibitory arrow*) but the mechanism is unclear. *GLIR* GLI repressor, *GLIFL* full-length GLI. **b** Non-canonical signaling via AMPK in muscle and brown fat. AMPK is activated by SMO via liver kinase complex B1 (*LKB1*) and calcium/calmodulin-dependent kinase kinase 2 (*CAMKK2*). In turn, adenosine monophosphate kinase (*AMPK*) represses cholesterol production directly by inhibition of HMG-CoA reductase (*HMGCR*) and sterol regulatory element-binding protein 2 (*SREBP2*) and indirectly through mTORC1 inhibition. In this context cyclopamine was reported to act as a partial agonist of SMO, but whether cholesterol activates SMO has not been tested (*question mark* above *arrow*). Lipid/sterol molecules and lipogenic transcription factors are shown in *red. Arrows* indicate activation (*arrowhead*) or inhibition (*orthogonal bars*), except for the arrow from PI to PI(4)P, which indicates an enzymatic step

Phosphoinositides also regulate the entry of SHH pathway negative regulators intraflagellar transport-A (IFT-A), GPR161, and Tubby-like protein 3 (TULP3) into the cilium [144–147]. The enzymatic activity of ciliary phosphoinositide 5-phosphatase regulates the ratio of PI(4)P and PI(4,5)P2 within the cilium shaft, maintaining a lipid composition that is responsive to modulation by PTCH1 [148, 149]. As these negative regulators are themselves excluded from the cilium following SHH binding of PTCH1, it is evident that multiple layers of SHH pathway repression are coordinated by PTCH1 via phosphoinositides. Together these data highlight the essential and diverse modes of SHH pathway regulation by lipids and lipid-modifying enzymes (summarized in Fig. 5a).

### Canonical signaling

Evidence is accumulating that signaling diverges downstream of SMO activation along canonical and non-canonical pathways. The canonical pathway is the best studied and is mediated by GLI transcription factors, which regulate the developmental patterning function of sonic hedgehog by either activating gene expression, via GLI1 and GLI2, or repressing it through GLI3 repressor (GLI3R) formation [150]. Lipid metabolic homeostasis has emerged as a critical function of hedgehog signaling in the liver, which is mediated by GLI proteins. Conditional deletion of SMO in mouse hepatocytes revealed that GLI1 and GLI3 coordinately repress lipid synthesis at the transcriptional level, presumably through an indirect mechanism that is not well-defined [151] (Fig. 5a). Furthermore, mutant livers displayed a metabolic shift of glucose utilization into the fatty acid synthesis pathway. Whether the regulation of lipid metabolism by canonical hedgehog signaling has functional significance during the formation of tissues dependent on hedgehog for their identity and structure has not been addressed.

### Non-canonical signaling

Evidence has begun to emerge that SMO activity reciprocally regulates lipid metabolism via a GLI-independent non-canonical pathway that is centered on adenosine monophosphate kinase (AMPK) [152]. AMPK regulates energy homeostasis within cells by switching off anabolic processes that consume adenosine triphosphate (ATP), including lipid synthesis, whereas it activates alternative catabolic pathways that generate ATP [153]. AMPK functions as an energy sensor through its binding of AMP in energy-deficient conditions, which promotes its activation by the upstream liver kinase complex B1 (LKB1) and calcium/calmodulin-dependent kinase kinase 2 (CAMKK2). In brown adipocytes, SMO activators including oxysterols stimulate rapid glucose uptake and aerobic glycolysis via AMPK that does not require GLI transcriptional activity [152]. These short-term metabolic changes are reinforced by a longer term GLI-mediated transcriptional response, resulting in extensive modulation of the cellular metabolic profile including lipid synthesis [152].

AMPK represses fatty acid, triglyceride, and cholesterol synthesis directly in several ways. Phosphorylation of acetyl-CoA carboxylase (ACC), a direct target of AMPK, inhibits the formation of malonyl CoA, the precursor for fatty acid synthesis [154]. In addition, AMPK directly represses the proteolytic processing, nuclear translocation, and transcriptional activity of SREBP1 [155]. Inhibition of sterol synthesis occurs through direct binding and phosphorylation of HMGCR [156] and SREBP2 [155], which in the latter case has broad-ranging effects similar to SREBP1 inhibition. The direct regulation of lipid metabolism is complemented by indirect inhibitory effects mediated by the mechanistic target of rapamycin complex 1 (mTORC1)-S6K kinase pathway, which in an opposite manner to AMPK is activated by nutrient availability and promotes anabolic processes, including lipid synthesis [157]. AMPK represses the activity of this complex by direct phosphorylation of mTOR and tuberous sclerosis complex (TSC) [158, 159], leading to reduced lipid synthesis (Fig. 5b). These data raise the possibility that a feedback loop involving cholesterol, SMO, and AMPK could modulate the output of non-canonical signaling to effect metabolic changes over short time scales. However, the functional significance of the reciprocal regulatory relationship between the hedgehog pathway and lipids remains unclear.

## Future directions in understanding the effects of metabolism on hedgehog signaling

The similarities in the phenotypes of inborn errors of sterol metabolism and SHH deficiency prompted investigation into how lipid metabolism and hedgehog signaling intersect. Despite intensive efforts, the involvement of lipids in hedgehog signaling arguably remains the most puzzling aspect of hedgehog signal transduction. A model of how lipids are involved in PTCH1 regulation of SMO is beginning to emerge, but lipid involvement in hedgehog signaling is complex and multifaceted. Nevertheless, findings to date, which are limited by the lack of in vivo analysis in higher vertebrates, suggest there is evolutionary conservation of the core mechanism. Cholesterol seems to be the main physiological agonist of SMO in higher organisms. The differences that have emerged between *Drosophila* and humans in the selectivity of the CRD for sterol binding partners and the evidence of alternative binding sites in SMO [112, 160] raises the question of what the physiologically relevant lipid binding interfaces of SMO are. Whether cholesterol and phospholipids mediate the effects of PTCH on SMO through distinct SMO domains will be important to explore further. Whereas cholesterol appears to act as an allosteric regulator of SMO, further studies are needed to determine whether PI(4)P has a similar function. A broader question relates to the dependence of endogenous SMO activation on diverse lipid molecules with positive and/or negative effects on signaling and whether these lipids act directly on SMO, or by modulating vesicular trafficking. Oxysterols appear to be of lesser importance under normal physiological conditions but in certain cancers associated with aberrant hedgehog signaling and dysregulated sterol metabolism, such as medulloblastoma, a role for oxysterols in promoting tumorigenesis via hedgehog signaling might have greater significance [107]. Finally, the relevance of the reciprocal regulation of lipid metabolism by canonical and non-canonical hedgehog signaling pathways is poorly understood and could prove to be important in tumor cells. Indeed, a transcriptional analysis of the response to SHH stimulation in cultured cells revealed alterations in metabolic pathways, including lipid metabolism, associated with invasive cancer [152], further highlighting the interaction between lipid metabolism and hedgehog signaling as fertile ground for future investigation.

## Abbreviations

7DHC: 7-dehydrocholesterol
ABCA1: ATP binding cassette subfamily A member 1
ABCG1: ATP binding cassette subfamily G member 1
ACAT: Acyl-coenzyme A:cholesterol acyltransferase
ACC: acetyl-CoA carboxylase
AMPK: Adenosine monophosphate kinase
ApoE: Apolipoprotein E
ATP: Adenosine triphosphate
BOC: Brother of CDO
CAMKK: Calmodulin-dependent kinase kinase
CDO: Cell adhesion molecule-related downregulated by oncogenes
CRD: Cysteine-rich domain
DHCEO: 3β,5α-dihydroxycholest-7-en-6-one
DHCR24: Desmosterol reductase gene
DHCR7: 7-dehydrocholesterol reductase
DHH: Desert hedgehog
DISP: Dispatched
ER: Endoplasmic reticulum
GAS1: Growth arrest specific protein 1
GPCR: G protein-coupled receptor
HHAT: Hedgehog acyltransferase
HMGCR: HMG-CoA reductase
HPE: Holoprosencephaly
ICD: Intracellular domain
IFT: Intraflagellar transport
IHH: Indian Hedgehog
INSIG: Insulin-induced gene protein
LKB1: Liver kinase complex B1
LXR: Liver-X receptor
mTORC1: Mechanistic target of rapamycin complex 1
NPC: Niemann-Pick C
OHC: Hydroxycholesterol
ORP: Oxysterol binding protein-related protein
OSBP: Oxysterol binding protein
PIP: Phosphatidylinositol-phosphate
PM: Plasma membrane
PTCH: Patched
RND: Resistance-nodulation-division
SAG: Smoothened agonist
SC5DL: Sterol C5-desaturase-like gene
SCAP: Sterol regulatory element-binding protein cleavage-activating protein
SCUBE: Signal peptide, CUB domain and EGF like domain containing
SHH: Sonic hedgehog
SLOS: Smith-Lemli-Opitz syndrome
SMO: Smoothened
SREBF: Sterol regulatory element binding transcription factor gene
SREBP: Sterol regulatory element-binding protein
SSD: Sterol-sensing domain
TMD: Transmembrane domain
TSC: Tuberous sclerosis complex
TULP3: Tubby-like protein 3
